# Mind the gap: residual malaria transmission, veterinary endectocides and livestock as targets for malaria vector control

**DOI:** 10.1186/s12936-015-1063-y

**Published:** 2016-01-12

**Authors:** Carlos Chaccour, Gerry F. Killeen

**Affiliations:** Department of Internal Medicine, Clinica Universidad de Navarra, Pamplona, Spain; ISGlobal, Barcelona Ctr. Int. Health Res. (CRESIB), Hospital Clínic, Universitat de Barcelona, Barcelona, Spain; Instituto de Salud Tropical, Universidad de Navarra, Pamplona, Spain; Ifakara Health Institute, Environmental Health and Ecological Sciences Thematic Group, Ifakara, Kilombero, Morogoro, United Republic of Tanzania; Department of Vector Biology, Liverpool School of Tropical Medicine, Liverpool, UK

**Keywords:** Ivermectin, Endectocides, Vector control, Residual transmission, Livestock, Zoophilic vectors, Malaria elimination

The work of Pooda et al. published in *Malaria Journal* [1] provides encouraging evidence of the potential use of systemic insecticides in cattle as a complementary means to further reduce residual malaria transmission that persists despite high coverage of current front-line vector measures, namely long-lasting insecticidal nets (LLINs) and indoor residual sprays (IRS).

LLINs and IRS interventions are responsible for most of the remarkable reductions in malaria burden achieved in this century [2], but even more ambitious new vector control measures will be required to achieve elimination of transmission from most endemic areas in the years ahead [3–5]. This is because LLINs and IRS leave two obvious spatial and temporal gaps wherever vector mosquitoes attack people outdoors, especially in the evenings and mornings, or rest outdoors before and after feeding [3–5]. There is, however, a third gap that does not usually receive as much attention, specifically their failure to kill mosquitoes that feed on animals rather than humans.

Zoophagic vectors that feed predominantly on animals can sustain malaria transmission even if they only bite humans infrequently [6]. Even with near-complete coverage of human sleeping spaces and houses, LLINs and IRS cannot be reasonably expected to have any meaningful impact upon the density or longevity of zoophagic vector populations, because they achieve no insecticidal coverage of the animals that constitute their main source of protein [4, 6].

Fortunately, by far the most common source of blood for most zoophagic malaria vectors are domesticated livestock, cattle in particular [7], so it is also possible to control the malaria transmission they mediate through veterinary applications of insecticides [8], the most exciting of which may be the systemic insecticides which the mosquito actually ingests along with its blood meal. Fritz et al. first described increased mortality of *Anopheles gambiae* feeding on ivermectin-treated cattle and suggested a potential role of this strategy for integrated vector management [9]. These findings have since been extended to *Anopheles culicifacies* and *Anopheles stephensi*, the main malaria vectors of Pakistan [10], and more recently to an important African vector of residual transmission, *Anopheles arabiensis* [11].

This latest report by Pooda et al. [1] now demonstrates similar increased mortality and reduced fertility of *Anopheles coluzzii*, a widely distributed vector species which maintains robust malaria transmission all across west and central Africa [12]. Interestingly, the lethal effect of ivermectin was seen even when the colony used had high prevalence of the *kdr* mutation which contributes to pyrethroid resistance in many parts of Africa.

Although the evidence base is growing fast, the full potential of ivermectin for malaria vector and transmission control remains to be established, but most discourse thus far has focused on medical delivery to human beings [13]. However, the alternative strategy of veterinary delivery to livestock has several advantages:

- Long-lasting injectable veterinary formulations of ivermectin already exist that can dramatically increase the effectiveness of this approach, by not only targeting a more important blood source for vector populations than humans, but also by achieving far longer duration of efficacy than is possible with the oral formulations available for human pharmaceutical delivery.
- A much greater diversity of different endectocides are available for cattle and other livestock, which offers an opportunity to combine drugs with different mechanisms of action, especially if ivermectin is to be used for mass drug administration to humans.
- Integrating an endectocide into traditional zooprophylaxis strategies [14] removes potential risks of accidentally increasing malaria transmission by increasing vector survival and reproduction [15], because mosquitoes attracted to feeding on animals could be killed rather than merely diverted away from humans.
- Endectocides can contribute to an overall *One Health* strategy by simultaneously improving livestock and human health. Nonzoonotic livestock parasites pose an important burden on human health by reducing economic output and nutrient availability. In addition to preventing malaria transmission, broadening the use of veterinary endectocides also offers an excellent opportunity to alleviate poverty and malnutrition by reducing the burden of livestock parasites on the health and economic resilience of their human owners [16].

*Plasmodium falciparum* and *Plasmodium vivax* are both strict anthroponoses, so it is understandable that ivermectin mass drug administration for malaria control and elimination is usually viewed primarily as an intervention for human populations. However, the use of veterinary antiparasitic drugs with insecticidal proprieties in domesticated livestock could perhaps achieve greater impact in many settings where persisting transmission is mediated by zoophagic vectors, and contribute to human health in previously unforeseen ways.

## Abbreviations

LLINs: long-lasting insecticide-treated nets
IRS: indoor residual spraying
